# Understanding the perceived role of electronic health records and workflow fragmentation on clinician documentation burden in emergency departments

**DOI:** 10.1093/jamia/ocad038

**Published:** 2023-03-11

**Authors:** Amanda J Moy, Mollie Hobensack, Kyle Marshall, David K Vawdrey, Eugene Y Kim, Kenrick D Cato, Sarah C Rossetti

**Affiliations:** Department of Biomedical Informatics, Columbia University, New York, New York, USA; Columbia University School of Nursing, New York, New York, USA; Geisinger Health Steele Institute for Health Innovation, Danville, Pennsylvania, USA; Geisinger Health Department of Emergency Medicine, Danville, Pennsylvania, USA; Department of Biomedical Informatics, Columbia University, New York, New York, USA; Geisinger Health Steele Institute for Health Innovation, Danville, Pennsylvania, USA; Department of Emergency Medicine, Columbia University Irving Medical Center, New York, New York, USA; Columbia University School of Nursing, New York, New York, USA; Department of Emergency Medicine, Columbia University Irving Medical Center, New York, New York, USA; Department of Biomedical Informatics, Columbia University, New York, New York, USA; Columbia University School of Nursing, New York, New York, USA

**Keywords:** electronic health record, emergency medicine, workflow, documentation burden, burnout, physician, nurse

## Abstract

**Objective:**

Understand the perceived role of electronic health records (EHR) and workflow fragmentation on clinician documentation burden in the emergency department (ED).

**Methods:**

From February to June 2022, we conducted semistructured interviews among a national sample of US prescribing providers and registered nurses who actively practice in the adult ED setting and use Epic Systems’ EHR. We recruited participants through professional listservs, social media, and email invitations sent to healthcare professionals. We analyzed interview transcripts using inductive thematic analysis and interviewed participants until we achieved thematic saturation. We finalized themes through a consensus-building process.

**Results:**

We conducted interviews with 12 prescribing providers and 12 registered nurses. Six themes were identified related to EHR factors perceived to contribute to documentation burden including lack of advanced EHR capabilities, absence of EHR optimization for clinicians, poor user interface design, hindered communication, increased manual work, and added workflow blockages, and five themes associated with cognitive load. Two themes emerged in the relationship between workflow fragmentation and EHR documentation burden: underlying sources and adverse consequences.

**Discussion:**

Obtaining further stakeholder input and consensus is essential to determine whether these perceived burdensome EHR factors could be extended to broader contexts and addressed through optimizing existing EHR systems alone or through a broad overhaul of the EHR’s architecture and primary purpose.

**Conclusion:**

While most clinicians perceived that the EHR added value to patient care and care quality, our findings underscore the importance of designing EHRs that are in harmony with ED clinical workflows to alleviate the clinician documentation burden.

## INTRODUCTION

Over the past 2 decades, emergency department (ED) visit volume has increased by more than 50% in the United States.^1^ Of significant and widespread concern, ED crowding—the inability of EDs to accommodate the demands of patient care given the resources available^2^—has been linked to poor patient outcomes and care quality, increased clinical errors, patient dissatisfaction, and clinician burnout.^2^ Disproportionally higher than other practice settings,^3^ 40%–60% of ED physicians^3^ and 25%–55% of ED nurses^4^ in the United States exhibit symptoms of burnout, with excessive documentation pinpointed as a mediating factor.^3^ EDs are characterized by the unpredictable patient census, fluctuating acuity levels, and persistent interruptions that shift priorities to optimize patient care, necessitating documentation that is comprehensive, precise, *and* timely.^5–7^ These ED characteristics contribute to high workloads placed on their clinicians.^8^

Evidence suggests that electronic health record (EHR) design and workflows fail to optimally meet the needs of ED workflows^9–11^—their incongruencies often leading to excess work and time-consuming workarounds.^11^ EHR configurations (eg, supporting regulatory compliance and quality improvement efforts) have intensified ED physician^9^^,^^12^^,^^13^ and nurse^14^ workload, particularly, noncare-related documentation. Consistent with Cohen et al,^15^ we define EHR documentation *burden* as additional work (ie, documentation or actions) performed in the EHR beyond that which is essential for “good” clinical care.^15^ However, documentation burden remains poorly understood and ill-defined, and no consensus exists on optimal approaches to scalably measure documentation burden using data sources leveraged from the EHR.^16^ Previously, we conducted a scoping review^16^ to synthesize existing literature on the manner in which documentation burden has been historically measured. We identified 7 *effort* constructs: *EHR usage and workload, clinical documentation and review, administrative tasks, cognitively cumbersome work, workflow fragmentation,* and *patient interaction*. How these constructs directly relate to EHR work remains unclear. Therefore, further qualitative research is essential to ascertain the perceived causes of EHR documentation burden and attributes of clinician EHR workflows that are worthwhile to study.^17^

*Workflow fragmentation* has emerged as one potential approach for evaluating ED EHR documentation burden.^18^^,^^19^ In a study conducted by Park et al,^11^ EHR implementation was associated with increased physician “time and attention” spent on charting, which exacerbated multitasking and care team interruptions. Often confounded for multitasking, which involves performing multiple tasks concurrently (eg, documenting and communicating),^18^ we define *workflow fragmentation* in the context of *task switching*, the observable alternation between 2 or more discrete tasks presented chronologically without overlap.^20–22^ According to the time-based resource-sharing model, *task switching* is an attention-sharing process involving “rapid and frequent switching between processing and maintenance [in working memory] that occurs during the completion of a task.”^23^ Task switching is often a consequence of interruptions and distractions,^6^ which contribute to cognitive fatigue and may lead to medical errors, threats to patient safety and care quality,^6^ and incomplete work.^24^

In the last decade, studies examining ED clinician workflows and workload in the context of EHR use largely have been conducted outside the United States.^25–28^ Concurrently, US-based research has predominately focused on the quantification of ED work using direct observation methods^9^^,^^10^^,^^13^^,^^14^^,^^29–32^ and retrospective data analyses,^33–36^ and on the use of surveys to assess ED clinician sentiment.^30^^,^^34^ Qualitative research on ED clinician EHR use has examined overall satisfaction, the effect of EHR usability and policy on clinical workflows,^12^^,^^37^ and their perceptions of EHR clinical documentation^10^^,^^11^; however, these studies exclusively highlight physician experiences. To our best knowledge, no qualitative studies have investigated the role of EHRs on clinician documentation burden among US prescribing providers *and* nurses. Moreover, there is a scarcity of research characterizing actionable EHR documentation burden-related challenges. The purpose of this study is to explore EHR factors that are perceived to contribute to clinician documentation burden and to understand the perceived role of *workflow fragmentation* on EHR documentation burden among ED prescribing providers and nurses.

## OBJECTIVE

The aim of this study was to understand the perceived EHR factors that contribute to clinician documentation burden and the perceived role of workflow fragmentation on EHR documentation burden among ED prescribing providers and nurses in the United States.

## METHODS AND MATERIALS

### Workflow fragmentation

No standard definition exists for *workflow fragmentation*. Consistent with Zheng and colleagues,^17^^,^^19^ we define *workflow fragmentation* as “the frequency of task switching and interruptions”. To qualitatively understand clinician experience, we operationalize *workflow fragmentation* in the context of task switching using the following constructs: (1) switching between EHR-mediated and non-EHR-mediated work (eg, work associated with the EHR vs direct patient care); (2) switching between EHR tasks; and/or (3) switching within EHR tasks (eg, note writing). We shared this definition with all participants during each interview (see Supplementary Material).

### Study design

#### Study setting and sample

From February to June 2022, we conducted a qualitative study to understand US ED clinician perspectives on the impact of EHR activities and workflows on clinical documentation burden through individual semistructured interviews. We targeted prescribing providers and registered nurses who actively worked in a US adult ED with the Epic Systems EHR (Epic Systems Corporation, Verona, Wisconsin). Study participants were included regardless of position (eg, staff nurse, case manager) and the proportion of time spent practicing in the ED. Ideal sample sizes vary based on the literature; however, similar studies^38–40^ suggested that a total of 12 registered nurses and 12 physician interviews would be sufficient to achieve thematic saturation.

#### Recruitment

We recruited participants through 3 channels: (1) ED- (eg, Society for Academic Emergency Medicine, American College of Emergency Physicians) and medical informatics-focused (eg, American Medical Informatics Association) professional listservs, (2) social media (eg, Twitter, LinkedIn, Facebook) which included a direct link to the Information Sheet, and (3) email invitations sent to and forwarded by clinicians, healthcare leaders, and colleagues (ie, purposive and snowball sampling). The recruitment emails included essential details about the study as well as the Information Sheet.

#### Interview guide

Semistructured interviews were steered by an interview guide (see Supplementary Materials) developed based on the results of our previously published scoping review^16^ and research in the ED.^18^^,^^19^ The interview guide encompassed 4 main themes: (1) typical and burdensome EHR clinical documentation activities; (2) burdensome EHR characteristics; (3) EHR-related workflow and task fragmentation; and, (4) the impact of patient acuity on EHR-mediated clinical documentation burden. We employed broad, open-ended questions to elicit wide-ranging responses.

#### Data collection

Forty-five to 60-minute interviews were conducted over HIPAA-compliant Zoom video conferencing software (Zoom Meetings, San Jose, California) by one author (AM) who also took field notes throughout the process. The interviews were scheduled at a time convenient for the participants. Interested clinicians were screened and verbally consented prior to being interviewed. After obtaining consent, participants were sequentially assigned a session identification number (ID) and sent a link via Zoom chat to anonymously enter their demographic information (eg, age, gender, role, and specialty) into an online form using Qualtrics software (Qualtrics, Provo, Utah). These demographic data were linked back to the transcripts based on the session ID (see Supplementary Material). All interviews were audio-recorded with the participant’s permission. Physician participants did not receive compensation; nurses were offered a $100 Amazon gift card. Data collection ended once we achieved thematic saturation (ie, no new themes emerged) among participants.^41^ All procedures were approved by the Columbia University Irving Medical Center Institutional Review Board.

#### Member checks

We actively conducted member checking during the interviews to verify the accuracy and credibility of our findings. Throughout the interviews, the interviewer synthesized and restated the content that participants shared within and between interviews to participants.

### Qualitative analysis

We followed the qualitative analytical approach prescribed in the Framework Method^42^: transcription, familiarization with interviews, coding, development of a working analytical framework, application of the analytical framework, data charting, and data interpretation. One author (AM) transcribed all audio-recordings verbatim using Microsoft Transcription Services, verified transcription accuracy, and updated transcripts for correctness; interview field notes were incorporated as appropriate.

We conducted inductive thematic analysis on the transcripts to identify prevalent themes that emerged in the interviews using NVivo software version 12 (QSR International, Burlington, Massachusetts). We define a theme as, “an abstract entity that brings meaning and identity to a recurrent experience and its variant manifestations […] captur[ing] and unif[ying] them into a meaningful whole”^43^. First, one author (the interviewer) independently and systematically reviewed the transcripts to become familiar with the data at a high level and assigned an initial code to semantically meaningful portions of text (ie, open coding) guided by our existing task taxonomy.^44^ We utilized open coding to identify additional and more granular categories. The coding scheme was updated based on new themes, subthemes, and categories that emerged, and refined in a second pass of the transcripts. Individual portions of text were coded with as many relevant themes that we saw fit. Preliminary themes and subthemes were presented to 2 coauthors (MH and SR) who independently reviewed and updated them based on the transcripts. The results were then discussed and recoded among these 3 coauthors (AM, MH, and SR) with informatics and clinical experience (ie, team-based open-coding^45^). Iteratively, the 3 authors achieved consensus in the analytical working framework and collaboratively finalized themes. Finally, all subthemes were categorized based on 6 domains described in the American Nursing Informatics Association (ANIA) *Conceptual Framework to Address the Burden of Documentation in the EHR*: reimbursement, regulatory, self-imposed, etc.^46^ (see Supplementary Table). Lastly, we synthesized information on shift structures and site characteristics that clinicians self-reported during the interview or follow-up communication.

## RESULTS

### Participant characteristics

We conducted 24 interviews among US physicians (*n* = 12) and nurses (*n* = 12); while we targeted study recruitment to all prescribing providers, our sample did not include nurse practitioners, physician assistants or resident physicians (Table 1). Most participants were from the Northeastern United States (92%) followed by the Midwest (*n *= 1) and West (*n* = 1). Among participants, the care setting was divided evenly between urban and rural areas; none practiced in a suburban area. All participants had ≥3 years of nontrainee clinical experience. Two-thirds of participants had 3–10 years of Epic Systems EHR experience, one quarter had ≥11 years, and the remaining had ≤2 years (*n* = 2). More than half the participants were female; among physicians, a majority were male (83%) while most nurses were female (92%). Participant age was well-distributed among physicians whereas most nurses were aged 25–34 years. Over 80% of participants self-identified as White, 3 as Asian, and 1 as Black or African American.

**Table 1.** Demographic characteristics of study population stratified by clinician type

	Physician	Registered nurse	Total
	*N* (%)	*N* (%)	*N* (%)
Total^a^	12 (100)	12 (100)	24 (100)
Age (years)			
18–24	0 (0)	0 (0)	0 (0)
25–34	2 (17)	10 (83)	12 (50)
35–44	4 (33)	1 (8)	5 (21)
45–54	2 (17)	1 (8)	3 (12.5)
55–64	4 (33)	0 (0)	4 (17)
65 and older	0 (0)	0 (0)	0 (0)
Gender			
Female	2 (16.7)	11 (91.7)	13 (54)
Male	10 (83)	1 (8)	11 (46)
Race			
White	9 (75)	11 (92)	20 (83)
Black or African American	1 (8)	0 (0)	1 (4)
Asian	2 (17)	1 (8)	3 (13)
Ethnicity			
Yes	0 (0)	0 (0)	0 (0)
No	11 (92)	12 (100)	23 (96)
Prefer not to say	1 (8)	0 (0)	1 (4)
Highest academic degree			
Associate degree	0 (0)	1	1 (4)
Bachelor’s degree	0 (0)	9	9 (38)
Master’s degree	0 (0)	2	2 (8)
Professional degree (eg, MD)	12 (100)	0 (0)	12 (50)
Doctorate degree	0 (0)	0 (0)	0 (0)
Role			
Staff nurse	n/a	12 (100)	12 (50)
Nurse practitioner	n/a	0 (0)	0 (0.)
Physician assistant	n/a	0 (0)	0 (0)
Resident physician	0 (0)	n/a	0 (0)
Fellow	1 (8)	n/a	1 (4)
Attending physician	11 (92)	n/a	11 (46)
US geographic region^a^			
Northeast	11 (92)	11 (92)	22 (92)
Central	0 (0)	1 (8)	1 (4)
Western	1 (8)	0 (0)	1 (4)
Southern	0 (0)	0 (0)	0 (0)
Care setting			
Urban	6 (50)	6 (50)	12 (50)
Rural	6 (50)	6 (50)	12 (50)
Suburban	0 (0)	0 (0)	0 (0)
Experience in current role (years)			
<1	1 (8)	0 (0)	1 (4)
1–2	2 (17)	2 (17)	4 (17)
3–5	0 (0)	4 (33)	4 (17)
6–10	3 (25)	5 (42)	8 (33)
11–20	3 (25)	0 (0)	3 (13)
21 or more	3 (25)	1 (8)	4 (17)
Total nontrainee clinical experience (years)			
<1	0 (0)	0 (0)	0 (0)
1–2	0 (0.0)	0 (0)	0 (0)
3–5	3 (25)	4 (33)	7 (29)
6–10	1 (8)	6 (50)	7 (29)
11–20	3 (25)	1 (8)	4 (17)
21 or more	5 (42)	1 (8)	6 (25)
Total Epic systems EHR experience (years)			
<1 year	1 (8)	0 (0)	1 (4)
1–2 years	1 (8)	0 (0)	1 (4)
3–5 years	3 (25)	5 (41)	8 (33)
6–10 years	3 (25)	5 (41)	8 (33)
11–20 years	3 (25)	2 (17)	5 (21)
21+ years	1 (8)	0 (0.0)	1 (4)

a Census regions of the United States.

All participants specialized in emergency medicine or nursing, while 2 participants, 1 physician and 1 nurse, additionally reported specialties in internal medicine and case management, respectively. More than half (*n* = 13) used only 1 EHR system in their clinical career, 7 used 2 (29%), and the remaining used >2 (17%).

The study sample represented urban and rural clinicians who worked across one or more of the following shift types: day, evening, and night, and one or more of the following shift lengths: 8-, 10-, and 12-hour; the majority worked 8- and 12-hour shifts. Urban and rural clinicians originated from large academic and nonacademic medical centers and community hospitals. Among rural clinicians, primary/critical access hospitals including single and dual prescribing provider EDs and those with <10 beds were sampled. Trauma Center Levels I–IV were represented across clinicians; however, only Levels I–II were represented among urban sites. All sites were nonprofit.

### EHR factors perceived to contribute to burden

**Figure 1.**
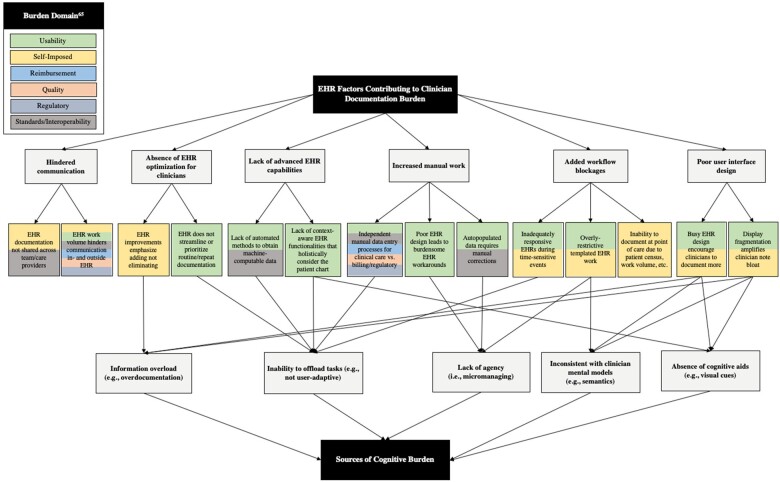
Thematic map of EHR factors contributing to clinician documentation burden and their relationship to identified sources of cognitive burden. Color coding aligns with ANIA documentation burden domains.

We uncovered 6 overarching themes associated with EHR features and functionalities perceived to contribute to clinician documentation burden (see Table 2 for descriptions): (1) advanced EHR capabilities are lacking; (2) EHR documentation is not optimized for clinicians; (3) EHR work volume hinders communication between clinicians internal and external to the EHR; (4) poor user interface design impacts clinician documentation habits; (5) high volume of manual EHR work; and (6) blockages in EHR impede documentation efficiency. Additional details on these themes and their subthemes (Figure 1) are described in Supplementary Material S3.

**Table 2.** Themes and subthemes on EHR factors perceived to contribute to clinician documentation burden

Theme	Subtheme	Description
Advanced EHR capabilities are lacking	Lack of context-aware functionalities in the EHR that consider the patient chart holistically	EHR functionalities are unable to fully account for patient content and context which leads to additional documentation and interactions that are irrelevant and/or erroneous to the encounter (eg, writing a comment to remove an irrelevant alert or issue that has already been addressed elsewhere in the chart). Because functionalities are not automatically tailored to actual patients’ needs and clinical presentation, clinicians are slowed down.
	Lack of intelligent and smart data capturing methods (eg, automated recording and processing of incoming data sources) to obtain machine-computable data	Manual entry of discrete data is burdensome and could be improved by intelligent and smarter data capturing methods (eg, voice capture and ambient documentation) to obtain machine-computable data. Discrete data capture, including checkboxes, may yield machine-computable EHR data, but do not provide an adequate, comprehensive clinical landscape and results in additional free-text documentation to supplement/fully describe the context; clinicians perceive this as less streamlined and redundant.
EHR documentation is not optimized for clinicians	EHR does not streamline or help prioritize documentation associated with hourly rounding or check-ins (eg, Q15)	EHRs do not sufficiently support the required repeat documentation that cyclically occurs in the ED (eg, hourly checks, intake and output, assessments, interventions such as restraints, etc.). This is especially burdensome when balancing critical and noncritical patients which may have the same documentation requirements that need to be entered on a recurrent basis.
	EHR improvements overemphasize adding, not optimizing or removing features	EHR development among vendors and EHR configuration among respective organizational leadership focus on adding more features in the EHR to manually capture more data from clinicians instead of finding better automated data capturing methods or solving underlying technological issues (eg, optimization of functionalities, better backend database models). The content is unrelated to direct patient care or good care quality.
Hinders communication between clinicians internal and external to the EHR	Volume of required EHR work hinders communication outside (eg, verbal orders, hands-on learning, and teaching opportunities) and inside the EHR (eg, lack of bandwidth to record high quality documentation)	Because clinicians are inundated with high volume EHR work, some information is not communicated in the chart. Thus, the burden of documentation gets shifted onto the next care team member. Simultaneously, volume of time spent on the computer documenting in the EHR impacts culture in the practice environment and reduces opportunities for hands-on learning and teaching between attendings and residents. Instead of direct, real-time communication between clinicians, clinicians must document everything in the EHR (for posterity purposes), which reduces efficiency and increases documentation long-term.
	EHR documentation not shared across team/care providers increases redundancies	The EHR is not an interactive, collaborative tool among clinicians and does not facilitate shared documentation that could be incrementally added between and across roles and specialties for the same patient. This often leads to redundant documentation across clinicians for the same encounter, as well as extraneous documentation between encounters across clinicians.
Poor user interface design impacts clinician documentation habits	Busy EHR design encourages clinicians to document more	Structured data fields, such as checkboxes, compel clinicians to document more out of the speed at which data could be entered, perceived medical legal necessity and/or lack of training. The ease in which structured fields could be documented in the EHR encourages clinicians to take shortcuts on the computer than to properly complete it at the patient bedside.
	Display fragmentation (Senathirajah et al^47^) amplifies clinician note bloat	Display fragmentation^47^ in the EHR leads to more documentation burden including note bloat because the same information is documented in multiple areas of the chart and/or data must be pulled into the note from different areas of the chart by the clinician in attempts to synthesize the data in one centralized place.
High volume of manual EHR work	Poor EHR design leads to burdensome EHR workarounds	EHR design that does not support clinician workflows leads to burdensome EHR workarounds. Because EHRs are unintelligent information systems that are unable to react promptly to new information, it is often easier (not necessarily more efficient) for clinicians to adopt strategies in the EHR to circumvent workflow blockages associated with design (eg, regulatory and reimbursement requirements, and quality metrics) than to remedy the underlying causes and circumstances in the healthcare system.
	Autopopulated data requires manual corrections	The EHR often autopopulates inaccurate or conflicting data (eg, completion timestamps that do not reflect when actual patient care was rendered) which require clinicians to manually review and update documentation post factum for correctness; these data may originate from other areas the EHR and/or systems that are not well-integrated into the EHR.
	Independent manual data entry processes for clinical care, and regulatory and billing requirements	EHRs do not capture billing, regulatory and reporting data effectively or efficiently as clinicians are required to manually enter these data as opposed to offering alternative data capturing methods (eg, ambient collection) which may be less disruptive to direct patient care. Lack of integration between data capture for patient and nonpatient care fragments the clinical workflow and increases the perception of double documentation (eg, capturing discrete events to compute a timestamp) among clinicians.
Blockages in EHR impede documentation efficiency	Overly restrictive, templated EHR work	While templated work in the EHR may standardize data entry, templated EHR work slows clinician documentation, and takes away individualized and critical thinking (ie, workflow blockades), and agency. With templated work, documentation is no longer based on medical/nursing expertise, but rather prescribed and restrictive, almost like hand holding; clinicians perceive this as robotic.
	Inadequately responsive EHRs in time-sensitive, critical events	Under critical circumstances where documentation is highly chronological and linear, the EHR is not sufficiently responsive or user-friendly, leading to double documentation. For example, during code blue and stroke events which involve rapid-fire execution of actions and documentation of information, clinicians (specifically nurses) default to using paper artifacts and/or back-charting due to concerns with charting accuracy and comprehensiveness.
	Inability to chart in EHR at point of care (eg, back-charting, work outside of work) due to patient census, work volume, and prioritization of direct patient care over documentation	Documentation in the ED must be both timely and thorough which adds to work pressures. Clinicians have the inability to chart at point of care due to high patient census and volume of work in the ED, and the prioritization of building rapport with patients and providing quality care over documentation. Specifically, work outside of scheduled shift (eg, note writing) is a significant issue among a subset of clinicians who are focused on communicating with patients.

### Perceived role of workflow fragmentation on EHR documentation burden

**Figure 2.**
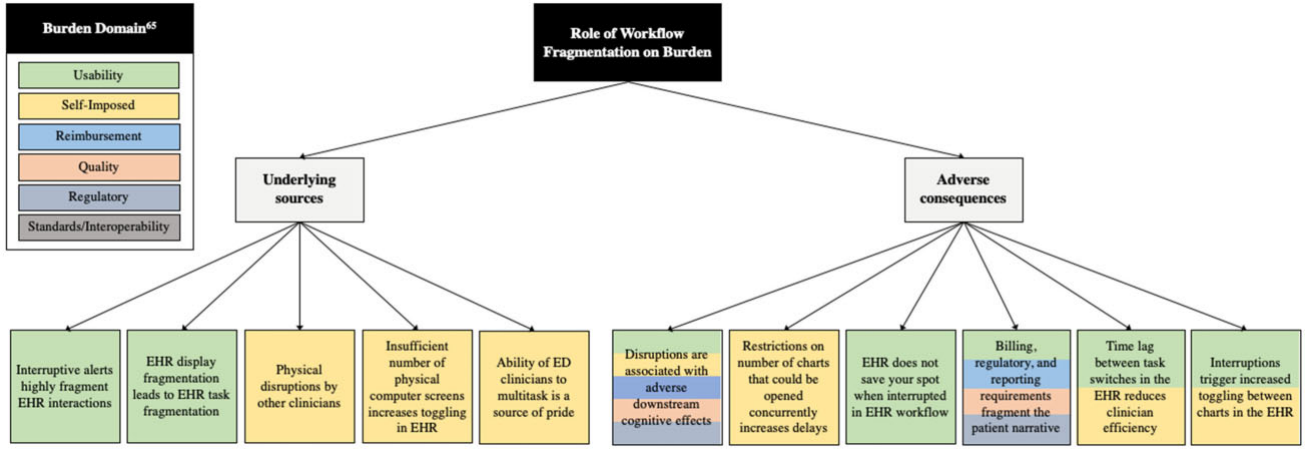
Thematic map of the role of workflow fragmentation on clinician documentation burden. Color coding aligns with ANIA documentation burden domains.

Two themes emerged on the perceived role of workflow fragmentation and EHR documentation burden (Table 3): (1) underlying sources: internal and external sources of workflow fragmentation including lack of peripherals, EHR design, physical disruptions, alerts, and organizational culture; and (2) adverse consequences: impact of EHR workflow fragmentation on clinician work including poorer documentation quality, reduced efficiency and detrimental cognitive effects (Figure 2). These themes and their subthemes are further elaborated in Supplementary Material S4.

**Table 3.** Themes and subthemes on the perceived role of workflow fragmentation on EHR documentation burden

Theme	Subtheme	Description
Underlying sources	Insufficient number of physical computer screens increases toggling within the EHR	Absence of 2 computer screens leads to increased toggling between screens, and between and within the EHR and other applications.
	EHR display fragmentation^47^ leads to EHR task fragmentation	Patient data is presented in disjoint areas of the chart and not synthesized or summarized for efficient consumption and information retrieval. This design flaw leads to more switching to perform a task.
	Physical disruptions by other clinicians	Physical disruptions between clinicians communicating in the ED while working are frequent. Interruptions are perceived as more salient when clinician is working in the EHR versus while providing direct patient care.
	Interruptive alerts highly fragment EHR interactions	Clinicians perceive interruptive alerts as occurring at the least opportune moments and become frustrated because it breaks up the flow of their work, particularly, when one must acknowledge an alert and respond to additional questions/add a comment.
	Ability of ED clinicians to multitask is a source of pride	ED clinicians take pride in being able to multitask; it is culturally ingrained and encouraged (ie, rating trainees) because ED clinicians treat multiple patients simultaneously that are not predictably organized into discrete clinical encounters.
Adverse consequences	Billing, regulatory and reporting requirements fragment the patient narrative story	Billing, regulatory and reporting requirements fragment the way one documents the patient narrative story (eg, clinicians generate separate notes for one clinical encounter to record separate timestamps).
	Restrictions on number of charts that could be opened concurrently increases delays	ED clinicians have large caseloads that may not be consistent with the institutional policy on maximum number of charts that could be opened simultaneously; therefore, clinicians need to open and fully close charts of active patients, adding more steps and time lag.
	Time lag between task switches in the EHR reduces clinician efficiency	Technological lag in having to switch in the system is a barrier to clinician efficiency and the perceived experience of inefficiency is cumulative over time.
	EHR does not save your spot when interrupted in the EHR workflow	Clinicians experience frustration when they leave their computer to attend to interruptions external to their EHR workflow, and their EHR session automatically times out and closes due to inactivity; any unsaved information entered in the chart is not automatically saved.
	Interruptions trigger increased toggling between charts in the EHR	Workflow fragmentation is additive, where interruptions drive additional switching in the EHR because they need to be processed and actioned for clinical care (eg, phone calls).
	Disruptions are associated with adverse downstream cognitive effects	Clinicians perceive that disruptions are associated with increased cognitive burden which all pose a safety issue. Task switching (ie, “running around”) and variety of documentation types (eg, assessments) in the ED leads to forgetting because clinicians cannot document at point of care, while the need to attend to interruptive alerts leads to loss of train of thought. Disruptions derail clinical thought processes because it is difficult to “[reconstruct] all the pieces”. Clinicians must reset their brains and ramp up cognitively postinterruption and increase their mental awareness to return to work after disruption.

### Perceived sources of cognitive burden in the EHR

We identified 5 themes on the perceived sources of EHR cognitive burden (Table 4): (1) inability to offload tasks; (2) absence of cognitive aids; (3) inconsistency with clinician mental models; (4); information overload; and (5) lack of agency. The relationship between these themes and those associated with EHR factors contributing to clinician documentation burden is outlined in Figure 1. Additional results are described in Supplementary Material S5.

**Table 4.** Themes on perceived sources of EHR cognitive burden

Theme	Definition in the context of cognitive load	Description
Inability to offload tasks (eg, not user-adaptive)	Offloading internal irrelevant processes (ie, tasks) onto external tools^48^	The EHR should adapt to the user’s needs and usage patterns as opposed to the user adapting to the EHR. For example, clinicians who are less computer literate or who are trainees may require different layouts and messaging (eg, clinical scoring tools) compared to more tech savvy, seasoned clinicians.
Absence of cognitive aids^49^ (eg, visual cues)	Tools that help prioritize tasks by reducing extraneous noise and enhancing the signal^49^	No visual cues in the EHR to help clinicians navigate to existing data that are relevant for the specific patient encounter.
Inconsistency with clinician mental models^50^ (eg, semantics)	Broad conceptualization of an individual’s thought process and how concepts interact in the world (eg, text structure)^50^	Inconsistency of language in the EHR with how clinicians discuss and think about medical concepts and terminology adds to cognitive burden of the EHR and slows clinicians down while navigating; as a result, they cannot find what they want to document in the chart.
Information overload^51^ (eg, overdocumentation)	Excessive data that hinders performance^51^	While there is a penalty for omitting data, there is no penalty for including too much data; this leads to overdocumentation (eg, lengthy notes, pulling additional data into notes). Overdocumentation makes it impossible for clinicians to review and retrieve relevant clinical information which adds to cognitive burden.
Lack of agency (ie, micromanaging)	Ability to control, predict and monitor one’s actions^52^^,^^53^	Clinicians perceive that rule-based EHR functionalities micromanage clinician work (eg, constant reminders and gatekeeping) at the least opportune moments, without having sufficient context of what is actually critical at the point of care.

## DISCUSSION

Over the last decade, EHR-related burden has emerged as a main driver of burnout among clinicians.^54^ Significant challenges remain in ascertaining the causes of clinician documentation burden such that solutions could be generated. In this study, we uncovered a wide breadth of themes that demonstrates the (between-topic) complexity of this domain but also consistency in the (within-topic) experience among clinicians. We identified 6 themes on EHR factors perceived to contribute to clinician EHR documentation burden based on interviews among a sample of US ED prescribing providers and nurses. Clinicians expressed that the EHR documentation burden emerged from several domains including *lack of advanced EHR capabilities*, *absence of EHR documentation optimized for clinicians*, and *poor user interface design*, which cumulatively impacted ED practice through *added workflow blockages, increased volume of manual work,* and *hindered communication*.

Many clinicians agreed that the EHR added value to patient care (ie, improved safety, interoperability, and information sharing). Clinicians speculated that the perceived burdensome characteristics associated with EHR design and configurations were an artifact of underlying systemic and external constraints including reimbursement, regulatory, and compliance requirements, in addition to medical-legal concerns, training, and organizational culture—all of which have been broadly acknowledged in the literature as exacerbating burden.^55^ Consistent with prior studies, clinicians collectively stated that EHR factors associated with poor interface design, usability, and user experience posed a major risk to patient safety.^37^ Given these results, it will be imperative to monitor how upcoming changes to the Evaluation and Management coding guidelines will impact clinician perceptions of the EHR.^56^

The relationship described between the EHR’s characteristics and perceived experience of burden among clinicians in our study indicates a continued need to design EHRs that are harmonious with ED clinical workflows. Participants expressed a demand for patient-context-aware EHR functionalities that adapt to individual clinical presentations to reduce irrelevant EHR interactions (eg, hard stops and extraneous documentation), smart data capturing methods (eg, automated recording and processing of incoming data sources) to obtain machine-computable data used for nonclinical purposes, EHR tools that anticipate and prioritize documentation, and streamlined EHR displays and configurations.^47^ Prior research has proposed that standardized and user-centered designs support ED workflows, and improve documentation efficiency and accuracy^37^; however, our findings suggest that overly restrictive, templated EHR workflows may contribute to clinician documentation burden and increase documentation inefficiencies. As opposed to hindering natural clinician workflows, and (by proxy) individualized thinking and medical expertise, clinicians stated that EHRs should adapt to individual users’ needs (eg, professional experience, technological literacy) and usage patterns. Therefore, thematic areas characterized in this study may be worthwhile to consider in the design and development of targeted interventions to relieve EHR documentation burden.

We found that EHR documentation burden *hindered communication* within and outside the EHR. Clinicians reported that the high volume of EHR work and EHR time spent deterred verbal communication and organic learning opportunities. They described reluctantly offloading redundant EHR work (eg, medication reconciliation) to the following clinician when overwhelmed by patient volume and critical care-related documentation. As a solution to eliminate documentation redundancies and unintended shifting of work among clinicians (within and across clinical encounters), participants considerably supported team-based care workflows and collaborative, incrementally added documentation. Similar findings can be found in the existing literature.^54^ These specific challenges may not require extensive EHR modifications as communication-related challenges are largely influenced by organizational culture (ie, “self-imposed”) and institutional decisions on EHR configurations. Nevertheless, given the breadth of the barriers identified, further stakeholder input and consensus will be essential to determine whether these perceived burdensome EHR factors could be addressed through optimizing existing EHR user interfaces and workflows alone, or if it will require a broad overhaul of the EHR’s underlying architecture (eg, database models) and purpose for which it was conceived (ie, patient care vs payment).

Clinicians indicated that automated EHR customizations (eg, user-adaptive EHR interfaces and functionalities) would be beneficial for attenuating the cognitive burden associated with EHR use (see Supplementary Material). Cognitive burden^57^ occurs when the pool of resources or “mental load” required to perform a working memory task^58^^,^^59^ exceeds an individual’s cognitive capacity to overcome its demands.^21^ Previously reported as a major source of frustration and burnout in the ED,^60^ we uncovered 5 themes that participants perceived to be linked to cognitive burden. Our results revealed that cognitive burden associated with EHR use and workflows emerged from multifaceted sources; these encompassed EHR system design including knowledge engineering (eg, inconsistent language describing medical concepts) and the user interface (eg, no visual cues to distinguish relevant information), to underlying data models (eg, rule-based EHR functionalities) that diminish clinician agency. Nearly all clinicians disclosed information overload as a leading source of cognitive burden. While regulatory and reimbursement requirements have been frequently implicated as the primary cause of EHR overdocumentation (eg, note bloat, redundant documentation),^61^ many clinicians expressed that overdocumentation was partly the result of their attempts to synthesize relevant encounter-related information recorded in disparate EHR regions in a centralized location.

Studies on EHR workflows indicate that task switching is salient in the ED,^18^^,^^19^ and that frequent task switching is correlated with elevated cognitive burden.^62^ In our study, clinicians perceived workflow fragmentation as relevant in their practice—particularly, task switching between EHR-mediated and non-EHR-mediated work (eg, external interruptions). Clinicians additionally expressed that task switching was deeply interwoven into their workflows, and therefore, an unconscious process while working in the EHR. We identified 2 themes on the perceived relationship between workflow fragmentation and EHR documentation burden: *underlying sources* and *adverse consequences*. Subthemes in these thematic areas were highly sociotechnical—alluding to the complex interplay among clinicians, the EHR, their physical workplace environment, organizational decisions, and cultural expectations in the ED—illustrating that EHR design alone is insufficient for resolving EHR-related burden.

According to participants, workflow fragmentation originated from physical disruptions by colleagues, inadequate computer peripherals (eg, single computer screen), and the EHR software (eg, interruptive alerts, display, and task fragmentation^47^). Described elsewhere in the literature as a cause of EHR cognitive burden,^63^ display fragmentation^47^ (ie, the need to click through several screens and views due to disjoint information), and task fragmentation^47^ (ie, “undesirable” separation of task components) in the EHR was reported as a design flaw and key driver of task switching among participants. Moreover, clinicians perceived that reimbursement, regulatory, and reporting requirements not only fragmented clinician documentation workflows but also resulted in poor documentation quality as it splintered the patient’s narrative. For example, 1 physician cited the need to author separate notes for pre- and postsedation assessments, which were distinct from the ED provider note, during one clinical encounter to log separate timestamps. Additionally, clinicians expressed frustration navigating through inefficiencies associated with task switches of varying etiology including institutional restrictions on the number of concurrently opened charts, increased toggling due to interruptions, technological time lags between task switches, and the inability of the EHR to automatically revert to timed-out sessions. A number of these sources of task switching may be related to “self-imposed” burden including infrastructure capacity (eg, server response time). Further evaluation is required to unravel whether these drivers of task switching are due to organizational decisions, EHR design constraints, or a combination of both.

Akin to existing frameworks that have characterized various documentation burden domains including organizational culture supporting more documentation (ie, “self-imposed”),^55^ we found that ED professional culture encouraged multitasking. Unsurprisingly, one of the primary ED challenges is simultaneously attending to several patients that are not structured into discrete clinical encounters. However, participants acknowledged that task switching posed a safety risk due to its associated cognitive burden, which often resulted in forgetting to document, loss of train of thought, and expending additional mental resources to return to work following disruptions, among other downstream consequences (Supplementary Material S4). These findings reinforce the need to address workflow fragmentation and to design and develop tools that not only facilitate effortless transitions between and within EHR tasks but also those that support seamless reintegration once a clinician is disrupted.

### Limitations

Due to the widespread experience of burden and burnout confronting ED providers across the United States, we recruited nationally as opposed to concentrating our efforts on one institution. While we achieved broad clinician representation across ED site characteristics (eg, urban/rural, academic/nonacademic, bed size, critical access, middle class/indigent populations, etc.), we acknowledge that our study sample is not representative of all practicing US ED physicians and nurses. Most participants originated from the Northeastern region of the United States with marginal representation from the West and Midwest; therefore, our results may not be generalizable to clinicians in the South and ED sites with characteristics that do not correspond with those represented among our participants (eg, small urban, suburban, freestanding, and for-profit EDs). Driven by the saturation of themes for our qualitative research objective, our study sample was adequate for our study design. Further work is required to verify the transferability^64^ of the study themes identified among targeted population samples.

Recruitment relied on professional listservs, social media, and purposive and snowball sampling. Furthermore, participants self-selected into our study, which may increase selection bias. Despite consistent research demonstrating that female physicians experience more EHR documentation burden compared to males,^65^^,^^66^ we only recruited 2 female physicians. Lack of representativeness among genders in our study may be associated with male physicians experiencing less burden (eg, time constraints) compared to their female counterparts. Additionally, we were unable to recruit nurse practitioners, physician assistants, and resident physicians who may document at drastically different volumes and/or follow distinct documentation patterns compared to attending physicians.

While the policy issues highlighted in the interviews were universal and salient throughout the participants, specific EHR features that clinicians referenced may be different between Epic Systems EHR instances within and across institutions and clinician roles. However, a strength of this study is that uncovered themes were consistent across roles, care population settings, institutions, and geographic regions. Lastly, as this study specifically targeted Epic Systems EHR users, the themes identified may not be generalizable to clinicians using other EHR systems (eg, Oracle Cerner, MEDITECH); participants who had experience with other EHR systems often pinpointed differences among systems. However, these EHR systems are out of scope for our study as we intend to use the interview results to inform analytical processes at our institution which utilizes Epic Systems EHR. Furthermore, inclusivity of other EHR systems may lead to overly heterogenous themes that are nonspecific and unactionable. For example, nearly all clinicians cited frustrations with the Epic Systems EHR sepsis best practice advisory.

### Future directions

We will harness themes uncovered in this study to drive our quantitative analysis of EHR documentation burden among ED clinicians using raw EHR audit logs and other EHR metadata with the overarching goal of developing a methodological pipeline to understand concepts associated with clinician workflow and operationalize burden. Concurrently, future efforts should consider targeted solutions among the EHR factors identified in our findings, prioritizing them into short-, medium- and long-term interventions.

## CONCLUSION

In this study, we aimed to understand the perceived role of the EHR and *workflow fragmentation* on ED clinician documentation burden. By and large, prescribing providers and nurses perceived that EHRs were conceptually and intellectually positive, adding value to patient care through improved safety, interoperability, and information sharing. However, clinicians also expressed that many EHR features and functionalities were a source of frustration, cognitive burden, and documentation burden, and therefore, a threat to patient safety and care quality. Our results demonstrate several areas of opportunity to improve EHR-related documentation burden and clinician workflow fragmentation, and a continued need to design EHRs that are harmonious with ED clinical workflows.

## Supplementary Material

ocad038_Supplementary_Data

## Data Availability

The disaggregate data underlying this study are not available in order to protect the privacy and confidentiality of the participants interviewed.
