# Supplementary material for: Understanding the perceived role of electronic health records and workflow fragmentation on clinician documentation burden in emergency departments
Source: J Am Med Inform Assoc. 2023 Mar 11;30(5):797–808. doi: 10.1093/jamia/ocad038 (PMC10114050; doi:10.1093/jamia/ocad038)
Supplement: ocad038_Supplementary_Data [file ocad038_supplementary_data.docx]

**Supplement 1. Semi-structured Individual and Group Interview Guide**

1. **Standard Demographic Questions**
2. Please enter your assigned session ID: ___
3. What is your age?
   1. 18-24
   2. 25-34
   3. 35-44
   4. 45-54
   5. 55-64
   6. 65 and older
4. What is your gender?
   1. Male
   2. Female
   3. Non-binary/third gender
   4. Prefer to self-describe (free text)
   5. Prefer not to say
5. What is your race? Do you consider yourself… (Select one or more.)
   1. White
   2. Black or African American
   3. Asian
   4. Native Hawaiian or other Pacific Islander
   5. American Indian or Alaska Native
   6. Other
   7. Prefer not to say
6. Are you Hispanic or Latino?
   1. Yes
   2. No
   3. Don’t know/not sure
   4. Prefer not to say
7. What is the highest degree or level of school you have completed?
   1. Associate’s degree
   2. Bachelor’s degree
   3. Master’s degree
   4. Professional degree (e.g., MD)
   5. Doctorate degree
8. What type of clinician are you?
   1. Registered nurse
   2. Advanced practice nurse
   3. Physician
   4. Other (please specify)
9. What is your role?
   1. Staff nurse
   2. Nurse educator
   3. Clinical nurse specialist (CNS)
   4. Nurse practitioner (NP)
   5. Nursing manager
   6. Nursing director
   7. Care coordinator
   8. Resident physician
   9. Fellow
   10. Attending physician (non-hospitalist)
   11. Attending physician (hospitalist)
   12. Other (please specify)
10. What is your specialty?
    1. Anesthesiology
    2. Dermatology
    3. Radiology (Diagnostic and Therapeutic) and Nuclear medicine
    4. Emergency Medicine
    5. Family Medicine
    6. Surgery (including General, Colorectal, Neurosurgery, Plastic Surgery, ENT, Orthopedic, Urology, and Vascular)
    7. Medicine (Internal medicine including specialties e.g., Oncology, Cardiology, etc.)
    8. Neurology
    9. Obstetrics and Gynecology
    10. Ophthalmology
    11. Pediatrics
    12. Rehabilitation
    13. Psychiatry
    14. Pathology
    15. Radiation Oncology
    16. Preventive Medicine
    17. Medical Genetics and Genomics
    18. Allergy and Immunology
    19. Other (Please specify)
11. Please select the setting(s) that you practice inn.
    1. Ambulatory
    2. Inpatient
    3. Other (please specify)
12. How many years of experience do you have in your current role?
    1. <1 year
    2. 1-2 years
    3. 3-5 years
    4. 6-10 years
    5. 11-20 years
    6. 21+ years
13. How many years of clinical experience do you have in total (not including student)?
    1. <1 year
    2. 1-2 years
    3. 3-5 years
    4. 6-10 years
    5. 11-20 years
    6. 21+ years
14. How many years have you been working with your current EHR?
    1. <1 year
    2. 1-2 years
    3. 3-5 years
    4. 6-10 years
    5. 11-20 years
    6. 21+ years
15. Please enter the total number of years that you have used each EHR system below for clinical care (enter zero if none)?
    1. Epic
    2. AllScripts
    3. Cerner
    4. MediTech
    5. McKesson
    6. Athena
    7. E-Clinical Works
    8. Practice Fusion
    9. Other: ________________________
16. **Interview Overview**

***Description*.** Thank you for agreeing to participate in this interview. We are interviewing you to better understand how electronic health record tasks and functionalities contribute to clinical documentation burden and burnout among clinicians in the emergency department. We invited you because you are a [registered nurse or prescribing provider] in the emergency department. We are interested in your experiences, so there are no right or wrong answers.

***Length.*** The interview should take approximately 45-60 minutes. You may stop the interview at any time.

***Permission to Start Recording.*** Do you have any questions? May I start recording?

Let’s get started…

1. **Group Interview Overview**

***Introductions.*** Hello, my name is [first name], and I’m a researcher at Columbia University. My work focuses on electronic health record documentation burden.

***Description*.** First, I want to welcome everyone. Thank you all for agreeing to participate in this research study. In this study, we would like to better understand how electronic health record tasks and functionalities contribute to clinical documentation burden and burnout among clinicians in the emergency department. We invited you because you are a [registered nurse or prescribing provider] in the emergency department. We are interested in your experiences, so there are no right or wrong answers.

***Length.*** The group interview should take approximately 45-60 minutes. You may stop the interview at any time.

***Ground rules.*** Before we get started, I want to set ground rules for this discussion today:

- Participation in the focus group is voluntary. You can leave the focus group at any point you wish.
- It’s okay to abstain from discussing specific topics if you are not comfortable.
- All responses are valid—there are no right or wrong answers.
- Please respect the opinions of others even if you don’t agree.
- Try to stay on topic; we may need to interrupt so that we can cover all the material. Speak as openly as you feel comfortable.
- We ask that you help protect others’ privacy by not discussing any of the details you hear today outside this group.

Does anyone have any questions about these rules?

***Permission to Start Recording.*** Are there any questions? May I start recording?

Let’s get started with a round of introductions, could you please say a few words about yourself: your name, your role, and if you were an egg how would you prefer to be cooked?

*Please note that this interview guide only represents the main themes to be discussed with the participants and therefore, does not include the various prompts that may also be used (examples given for each question). Non-leading and general prompts will also be used, such as “Can tell me a little bit more about that?” and “Do you have any other thoughts or comments?”.*

**Interview Questions (Example probes below questions)**

***Introduction questions (Establishing rapport for individual interview only)***

1. How long have you been working at your institution? What is your role?
2. Are you a trainee? What is your year?

***Typical and burdensome clinical documentation activities***

1. Could you describe the role of the electronic health record in your typical clinical workflow once a patient arrives in the emergency department?
   1. What types of electronic health record tasks are involved in each of these activities?
2. Clinicians and other healthcare leaders have different ideas about what clinical *documentation burden* means and what is the role of electronic health records…
   1. What does clinical documentation burden mean to you?
   2. Tell me your thoughts on how the electronic health record impacts your clinical documentation. (*If needed, clarify if it helps or hinders*)
3. Can you tell me about clinical documentation activities in the electronic health record that particularly hinder your clinical workflow?
   1. What characteristics about these activities make them particularly burdensome (e.g., does a task have a larger share of alerts compared to others)?

***Burdensome characteristics of the electronic health record***

1. Some define burden as added work or extraneous actions performed in the EHR beyond that which is required for good clinical care (i.e., additional work without any added value to good clinical care). Thinking about your last burdensome clinical shift…
   1. How did you interact with the electronic health record? (*If needed, ask to think about a shift in the past 2 weeks*)
   2. What did you do in the electronic health record that felt burdensome (e.g., write a note, retrieve information, interact with inbox messages, messaging with staff)?
2. What specific features of the electronic health record impede your clinical documentation workflow (e.g., alerts, design, screen-switching)?
   1. What makes [feature] particularly burdensome?
   2. Do you feel this often? (*If needed, clarify how frequent they experience this problem*)

***Workflow and task fragmentation in the electronic health record***

1. Through the literature, we identified several constructs that researchers have applied to measure clinical documentation burden in the electronic health record—one of which is *fragmentation of workflow*. We define *workflow fragmentation* in the EHR as the following: (a) switching between EHR-mediated and non-EHR-mediated work, (b) switching between tasks in the EHR, and/or (c) switching within tasks in the EHR. Thinking about what impedes your clinical documentation workflow and individual tasks in the EHR…
   1. How relevant is workflow fragmentation to you? What types of workflow fragmentation do you experience in the EHR?
   2. What characteristic(s) make EHR workflow fragmentation appropriate for measuring burden?
      1. In examining switching between EHR-mediated and non-EHR-mediated work…
      2. In examining switching between tasks in the EHR…
      3. In examining switching within tasks in the EHR…
   3. How does EHR workflow fragmentation hinder the optimal workflow for the user (e.g., interruptions)?
2. We also identified four constructs for time. How does the concept of time contribute to your experience of burden? (*If needed, clarify if the experience is there is burden task specific, in the overall day, urgency, or pace etc.*)
3. If you were to develop a measure of clinical documentation burden in the electronic health record, what would you include?

***Impact of patient acuity on EHR-mediated clinical documentation burden***

1. Clinicians have different preferences and patterns for completing the same type of clinical documentation in the electronic health record; for example, some clinicians in the ambulatory setting complete their documentation after their shift ends. How do you typically complete your clinical documentation in the electronic health record?
   1. Do you complete all your clinical documentation in the electronic health record for one patient in one instance? (*If needed, clarify if their documentation is consecutive and linear or if they document as they go*)
   2. Is there workflow fragmentation in how one patient encounter is documented in the EHR? How so?
2. Does your experience of documentation burden in the electronic health record vary between patients? How so?
   1. What about by patient acuity? How? (*If needed, clarify if there are typical workflows associated with patient acuity in the EHR. If so, are there some workflows that are more burdensome than others.*)
   2. What about emergency severity index? How?
3. How does patient case mix play a role in documentation burden in the electronic health record? (*If needed, clarify if there is a difference if they get three very ill patients and two moderately ill patients*)
   1. Are there different EHR-mediated clinical documentation workflows for different acuity levels?

***Closing statements***

1. Is there anything else that you would like to share that I haven’t already asked you about?

*Thank you very much for your time and the information you shared today. This is the end of the interview.*

********************************************************

**Additional Interview Materials (if required)**

| **Ten Constructs Associated with Measuring Burden** | **Four Constructs Associated with Quantifying Time** |
| --- | --- |
| 1. Electronic health record usage and workload 2. Clinical documentation and review 3. Electronic health record work afterhours and remote work 4. Cognitively cumbersome work (e.g., multitasking) 5. Administrative tasks (e.g., inbox management) 6. Fragmentation of workflow 7. Patient interaction | - - - 1. Average time spent       2. Proportion of time spent       3. Timeliness of completion (binary)       4. Activity rate |

**Supplement 2. Six domains of burden described in the ANIA Conceptual Framework for Addressing the Burden of Documentation in the EHR^65^**

| **Domain** | **Definition** |
| --- | --- |
| Reimbursement | Payment-related documentation and data entry |
| Regulatory | Accreditation-related documentation requirements |
| Quality | Documentation needed to validate that high-quality patient care was delivered |
| Usability | Additional time spent on EHR interactions due to poor human factors engineering and design |
| Interoperability/standards | Duplicated data entry due to poor configuration standards |
| Self-imposed | Organizational cultural influence on documentation habits |

**Supplement 3. EHR factors perceived to contribute to burden**

I. Advanced EHR capabilities are lacking

According to clinicians, *lack of context-aware functionalities in the EHR that holistically consider and account for the content in the patient chart* increased documentation and EHR interactions (e.g., clicks) that were not relevant and/or erroneous to the patient encounter. Because EHR functionalities were not automatically tailored to patients’ needs and their clinical presentation, clinicians were often slowed down by alerts which required additional work (e.g., writing comments) to action and remove workflow blockages [e.g., sepsis best practice advisory (BPA)], or to resolve issues that were already addressed elsewhere in the chart (e.g., prompts to order a COVID-19 test even if the patient had already tested negative during the clinical encounter). For instance, one nurse described, “if [a COVID test] comes back negative, it will still prompt us to order the COVID test, even though it has already been [completed]. And every time we log, we click into the chart, it comes up and you can't do anything… and it makes you write a comment also”. Referring to the indiscriminate nature of sepsis BPAs, another nurse indicated, “I'm a healthy 25-year-old with minimal medical problems. If I ran down the street and I got a cut on my finger, and […] they document my blood pressure being slightly elevated […], breathing a little too fast because I just ran down the street, it would be like, ‘sepsis alert’”.

Additionally, *lack of intelligent and smart data capturing methods*, such as tools that ambiently record and process incoming data for obtaining machine-computable data was cited as exacerbating documentation burden. One physician stated, “I think the biggest concern within the documentation burden is the requirement for discrete data and whether we can find smarter ways of entering that discrete data”. While manual data entry among clinicians (for discrete data capture) including checkboxes, potentially yielded machine-computable data in the EHR, they did not provide a comprehensive clinical picture, and thus, resulted in additional free-text documentation to supplement context; clinicians perceived this process as less streamlined and redundant.

II. EHR documentation is not optimized for clinicians

Documentation in the EHR has not been optimized for clinicians. Specifically, *EHRs do not streamline or help prioritize documentation associated with hourly rounding or check-ins (e.g., Q15 safety checks)*. As such, EHRs did not sufficiently support the required repeat documentation that cyclically occurred in the ED [e.g., hourly checks, intake (I) and output (O), assessments, interventions such as restraints, etc.]. This was especially burdensome when balancing critical and non-critical patients which required the same recurrent documentation:

“[I]t’s just very hard […] constantly putting in what the patient is doing at that moment, the hourly checks, the I’s and O’s, because if they’re a stable patient and they’re just here for arm pain. I can’t continually document when they’re going to the bathroom, when they’re eating because I’ve got a patient that is much sicker”.

Furthermore, *EHR improvements overemphasize adding, not optimizing or removing features* from the EHR. Historically, EHR development among vendors and EHR configuration among organizational leadership focused on injecting more features into the EHR to manually capture additional data unrelated to direct patient care or good care quality from clinicians, instead of improving automated data capturing methods or solving underlying technological issues (e.g., optimization of functionalities, improved backend database models). One physician elaborated, “somebody thinks, ‘OK, we just need the provider to do this one more thing, if they could just click this button or do this one more thing in Epic then we’d have this data and we could record off of it and do whatever’ and so sometimes it just gets piled on”.

III. Hinders communication between clinicians internal and external to the EHR

Because clinicians are inundated with a high volume of EHR work and do not have sufficient time to document on their shift, *communication inside (e.g., lack of bandwidth to record high quality documentation) and outside (e.g., verbal orders, hands-on learning, teaching opportunities) the EHR suffer*. Due to excessive EHR work, information was often not communicated in the EHR, which resulted in the shifting of the burden of documentation onto the next care team member. A nurse shared, “in triage it's more concise because I don't have that much time for patients […]. […] I don't have enough time to put the actual story, so I'm more likely to just verbally tell the doctor, but then of course that creates more time because then the doctor goes in”. Simultaneously, volume of time spent on the computer documenting in the EHR impacted practice environment culture and reduced opportunities for hands-on learning and teaching between attendings and residents because, “instead of a group of healthcare professionals that used to communicate a lot and have very good exchanges at the desk, now it's a desk of a bunch of people whose noses are buried in the computers […] to put information into the computer”. As opposed to relying on direct, real-time communication between clinicians such as, “yell[ing] out orders to the nurse” to “be done with it”, clinicians were required to “put in all kinds of other data” in the EHR, which reduced efficiency and time at the bedside.

Secondarily, a prevalent subtheme that emerged among a large subset of clinicians was that *EHR documentation was not shared across the patient care team and other care providers*, *which clinicians perceived as increasing redundancies*. Among clinicians, the EHR was perceived as neither interactive nor collaborative; particularly, the EHR did not facilitate patient documentation that could be incrementally added and shared between and across roles and specialties. This often led to redundant documentation across clinicians for the same clinical encounter, as well as extraneous documentation between encounters. Specific mention to *shared* and *interactive* documentation was noted among physicians, “I think a better approach would be to have […] a physical exam activity that's longitudinal and you see your [obstetrician gynecologist] and they put in the parts of the co-exam that are appropriate to their visit […]—a shared physical exam activity that is shared across providers across encounters that you can add to incrementally”, as well as among nurses, “I kind of wish that [the triage documentation] was a shared doc[ument] that you could see easier when a physician just asked the patient the same question and answered it. I wish it was more interactive”.

IV. Poor user interface design impacts clinician documentation habits

Many of the clinicians felt that the *busy EHR design encourages clinicians to document more.* Specifically, structured data fields, such as checkboxes, compelled clinicians to document more out of the speed at which data could be entered. The checkboxes also projected an impression of medical-legal necessity especially among those who lacked training. One nurse stated, “there seems to have been a shift recently to [nurses] becoming very task-oriented, and if there's a box, you click it. If there's a task, you click it. I, on the side, I teach and I've noticed a lot of my students feeling like, ‘well, the doctor didn't tell me to do that’ or ‘well, how would I know that I should assess that’ and it's sort of this lack of critical thinking, and so I think when there's a box to click, they expect it to be clicked because they want to justify what they've done”. Additionally, clinicians believed the ease in which structured fields could be documented in the EHR encouraged clinicians to take shortcuts on the computer rather than to do it correctly at the patient bedside:

“[M]ost people that use these checkboxed electronic health records with this, ‘check, check, check, check, check’ […]. [T]he record drives you to just say ‘yes’ or ‘no’ on all these questions […]. [T]hey're just doing it because that's the way the system is set up and it drives people to do things that are not correct, but it's the easy way, because to do it the right way you'd have to leave the computer, go back to the room, check those things before you went and actually put that stuff in the boxes”.

Another common subtheme among clinicians was that *display fragmentation in the EHR as defined in Senathirajah et al.,^48^ amplifies clinician note bloat.* Display fragmentation led to more documentation burden because patient information was documented in multiple areas of the chart (e.g., orders, vital signs, documentation, results, patent history, etc.). These scattered data points were pulled into the note from their respective EHR sections by the clinician in attempts to synthesize information in one centralized location. According to one clinician, “there's definitely fragmentation between test results and the documentation piece, and vital signs and all of this. And I think a lot of times, we try to solve that by pulling in all of that data into the notes so that it feels less fragmented, but then, I think it adds more bloat and kind of burden to having to read that note […]. [I]t doesn't add a lot of value, it just adds more bloat and challenges for people to try to read through it”.

V. High volume of manual EHR work

Among physicians and nurses, *poor EHR design leads to burdensome EHR workarounds.* Because EHRs were unintelligent information systems unable to react suitably and promptly to new information, it was often easier (not necessarily more efficient) for clinicians to adopt EHR strategies to circumvent workflow blockages associated with a flawed design related to regulatory and reimbursement requirements, and quality metrics. For instance, ostensibly straightforward actions such as deleting information inadvertently documented on the “wrong patient” or “wrong section… [was] difficult” for clinicians. One nurse said that while “[t]here's an option to cancel… it just kind of goes, reverts back to your originally saved. So a lot of times, if you enter something extraneously, you have to go and then add a note saying that you entered on the wrong patient or disregard and delete things rather than just a very quick, easy, just erase it all at once”. Clinicians expressed frustration that acquiescing to EHR design that did not support clinician workflows was easier than remedying the underlying cause and circumstances in the healthcare system:

“[T]he EHR has made time metrics really easy to measure and so because time metrics can be measured easily, they have become this all-consuming obsession of administration, which leads like any of these things to a nonsensical gaming of the system… So often, these time metrics lead to these bizarre side effects as people try to meet the arbitrary, where treating the metric becomes more important than treating the patient and leads to strange things”.

Another significant subtheme identified in the interviews was that *autopopulated data requires manual corrections.* The EHR often autopopulated inaccurate or conflicting data (e.g., completion timestamps that do not reflect when actual patient care was rendered) that required clinicians to manually review and update documentation post factum for correctness, “I would say that really does contribute to burden like things that don't even make sense, and it almost auto-documents something for you and then you have to go back and change it”. Additionally, these data originated from other areas the EHR and/or systems that were not well-integrated into the EHR, “it's set up so that each of those things has to be corrected manually, and so it's not designed to be intelligent, where it communicates with another piece of […] software”.

*Independent manual data entry processes for clinical care, and regulatory and billing requirements* was a key source of dissatisfaction across all clinicians. EHRs did not capture billing, regulatory and reporting data effectively or efficiently and required clinicians to manually enter these data which felt separate from patient care-related information:

“[D]ocumentation requirements don't align well with clinical care, and one example of that is […] for billing purposes. We are requested to document a certain number of review systems, but in an ED visit, even if you're taking extremely comprehensive care of a very sick patient, a 10-system review of systems may not be relevant or applicable”.

Likewise, clinicians expressed the EHR did not offer alternative, non-manual data capturing methods such as ambient collection which may be less disruptive to direct patient care. Meanwhile, lack of integration between data capture for patient and non-patient care fragmented the clinical workflow and increased the perception of double documentation (e.g., capturing discrete events in order to compute a timestamp) among clinicians.

VI. Blockages in EHR impede documentation efficiency

While templated work in the EHR may standardize data entry, several clinicians believed that *overly restrictive, templated EHR work* slowed their documentation, and detracted from individualized and critical thinking (i.e., workflow blockages), and agency. One nurse shared, “in reality and in practice, what you see is so much often not textbook, so you may have areas in that assessment, a pulmonary assessment, you say, ‘oh, but I also want to document this’… it’s meant to be streamlined, but it almost feels like taking the brain out of it—if that makes sense—taking the critical thinking out of it”, while a physician articulated, “there’s always nuance and I do worry about the customizability of the EHR becoming sometimes a crutch, sometimes a potential blockade to individualized thinking”. With heavily templated work, clinicians felt documentation was no longer based on medical/nursing expertise, but rather prescribed and restrictive, likening it to “hand holding”; clinicians perceived this as a robotic process.

Specifically, nurses reported *inadequately responsive EHRs in time-sensitive, critical events* as a major source of documentation inefficiency. Under critical circumstances where documentation was highly chronological and linear, the EHR was not sufficiently responsive or user-friendly, leading to double documentation. For example, during code blue and stroke events which involved rapid-fire execution of actions and documentation of information, many nurses described defaulting to paper artifacts and/or back-charting due to concerns with charting accuracy and comprehensiveness:

“I always advise somebody to take a piece of paper and whenever you hear something, just write it down and write down the time because you can always go back, find the time that you did it, and then just cross it out one by one so you don't miss anything”.

Another nurse stated:

“I know that this whole thing will have to be documented, so I try to keep if I can, I'll grab a piece of paper towel or something and I will write a little note… I’ll write more of a timetable”.

Lastly, nearly all clinicians express the *inability to chart in EHR at point of care (e.g., back-charting, work outside of work) due to patient census, work volume, and prioritization of direct patient care over documentation.* Clinicians shared that documentation in the ED must be both timely and thorough which added to work pressures. The could not chart at the point of care due to high patient census and volume of work in the ED:

“I’ve been so busy… I haven't gotten to even write the triage note. Sometimes I have to go in and backtrack the triage note, which is so bad of me. I'm like, ‘How does anyone even know anything about the patient if I haven't had the triage note down for hours?’”.

They voiced frustration in the deprioritization of rapport with patients and providing quality care over documentation. Specifically, work outside of scheduled shift (e.g., note writing) was a significant issue among a subset of clinicians who were focused on communicating with patients; for example, one clinician stated, “I almost never get my notes done on shift… I'm much more of a communicator. I'll go check on that patient rather than spend that 5 minutes getting caught up on all my documentation […]. I do find that people that are very good at getting their notes done, they definitely lose that human factor and I think that's huge. And for me, I just haven't been willing to give up that human factor, which means that I come in on my days off and get my notes done.”

**Supplement 4. Perceived role of workflow fragmentation on EHR documentation burden**

I. Underlying Sources: internal and external sources of workflow fragmentation

Clinicians reported five *internal and external sources of workflow fragmentation* in the ED clinical practice environment when prompted about task switching. *Insufficient number of physical computer screens* increased toggling between screens, and between and within the EHR and other applications:

“[S]creen switching can be a little bit difficult… we only have one screen so you've got, the EHR open, you have the web browser for our secure messaging so you can text other people open. If you want to review imaging, your EKG's, you have extra windows for those open”.

*EHR display fragmentation (a design flaw) drove EHR task fragmentation (i.e., task switching)* as patient data were presented in disjoint areas of the chart and not synthesized for efficient consumption and information retrieval:

“[A] lot of times we try to solve that by pulling in all of that data into the notes so that it feels less fragmented, but then I think adds more bloat and kind of burden to having to read that note”.

*Physical disruptions between clinicians* communicating in the ED while working were frequent and were perceived as more salient when the clinician was working in the EHR than while providing direct patient care:

“[T]hat's life in the ER… you're working on a note or you're putting orders in on one patient, and all of a sudden the nurse comes to talk to you about another patient. And oh, ‘hey Doctor [name], can you do this?’ And, ‘Oh yeah, alright let me click over let me click out of this thing that I'm doing and go over to this thing and put this order in’ and then I will say sometimes I forget what I was doing initially.

*Interruptive alerts highly fragmented the EHR interaction*, particularly, when one acknowledged and responded to additional questions associated with an alert or added a comment which clinicians perceived as occurring at the least opportune moments:

“[Y]ou're trying to work through and get this thing done and put these orders in and take care of this patient and then you [braking sound] get stopped and you have to make a comment about an allergy or you or something along those lines.”

Lastly, *perceived ability of clinicians to multitask was historically valued among ED prescribing providers* as indicated in the following quote:

“[W]e used to rate our residents on multi-tasking and what's been that term is all almost a bad term these days because we used to talk about and try to teach people to be proud of multitasking”.

II. Adverse consequences: poorer documentation quality, reduced efficiency, and detrimental cognitive effects

All participants described several subthemes in which workflow fragmentation was associated with poor documentation quality, reduced efficiency, and detrimental cognitive effects. First, *clinicians felt that billing, regulatory and reporting requirements fragmented documentation of the patient narrative story.* At some institutions, clinicians authored separate notes so that independent timestamps could be recorded for billing:

“[The] ED provider note is mostly supposed to be done before you sedate them, then you're supposed to do this pre-sedation assessment note, and then you have to do a post-sedation assessment note, and technically, within Epic you could do that all within the same note, but you have to go out and create these separate notes, which fragments the kind of the story and what happened during the encounter […] simply so that they're separate notes with separate timestamps on it”.

Many clinicians cited *restrictions on number of charts that could be opened concurrently contributed to workflow fragmentation and increased delays* in the EHR. As one participant indicated:

“[Y]ou can't have all of [your charts] open at once, so if you want to go to another one, you have to go back to your track board, click on the one you want, and then choose which one you want to exit out of. And if you click on the wrong person that you want to exit out of, then you're repeating the process over again. You can't quickly switch between the two”.

In general, institutional policy on the maximum number of charts that are allowed to be simultaneously opened among clinicians were not consistent with caseloads traditionally found in the ED at any given moment; therefore, clinicians continually opened and closed active patients’ charts when documenting, adding more steps and time lag in the EHR. Furthermore, the *time lag between task switches in the EHR reduced clinician efficiency*. This perceived experience of inefficiency due to the technological lag related to task switches in the system was cumulative over time:

“I don't know if this is necessarily EHR related or not, but it's the, sort of the speed or quickness of the EHR [...]. Maybe it's a computer system thing. Maybe it's an EHR thing, but oftentimes it's very frustrating when you can't move through the process as fast as you want to because the next screen pops up a couple seconds slower than you like to… I mean, it sounds silly or a fraction of a second slower than you want to, but those little fractions of a second really build up”.

Clinicians communicated experiencing frustration when they walked away from their computer to attend to interruptions external to the EHR, and their EHR session automatically timed out and closed due to user inactivity, or when another user logged in; distinctively, the *EHR did not save one’s spot when interrupted* in the workflow and any unsaved information entered in the chart was not automatically saved:

“[W]e all share workspaces, so if you get out of your computer, it doesn’t pull you back up in the same spot you were in... so when it does that, you’re kind of just going back to see what you reviewed last and trying to hope that you’re in the same spot that you walked away in the middle of... it’ll save the information without you hitting save. I’m so used to hitting save but it will save it, but it doesn’t show you where you stopped and you could be in the middle of a flowsheet.”

Additionally, *interruptions triggered increased toggling between charts in the EHR.* Thus, workflow fragmentation was described as additive where interruptions drove additional switching in the EHR because they needed to be processed and actioned for clinical care such as, “phone calls and communication that need to occur while in the EHR that are unrelated to those tasks” which required “an amount of switching between patient charts to process some of that information” that arrived. Lastly, clinicians were aware that *disruptions were associated with adverse downstream cognitive effects,* “lead[ing] to errors and distractions”. Clinicians perceived that disruptions were associated with increased cognitive burden which posed a threat to patient safety and care quality. Specifically, clinicians reported that task switching (i.e., “running around”) and variety of documentation types (e.g., assessments) in the ED led to forgetting because clinicians could not document at the point of care. Meanwhile, the need to attend to interruptive alerts led to loss of train of thought. Disruptions derailed clinical thought processes because it was difficult to “[reconstruct] all the pieces” they were working on pre-disruption. Post-disruption, there was a need to reset their brain, cognitively ramp up, and increase mental awareness to return to work.

**Supplement 5. Sources of cognitive burden in the EHR**

Throughout the interviews, clinicians alluded to five sources of EHR cognitive burden. Fittingly, these sources aligned with constructs that have been widely researched due to their relationship with cognitive load: (1) *inability to offload tasks*^64^; (2) *absence of cognitive aids*^59^; (3) *inconsistent with clinician mental models*^60^; (4) *information overload*^63^; and (5) *lack of agency.*^61,62^ As opposed to clinicians self-adjusting to the EHR, participants elaborated that EHRs should have the capacity to *adapt to user’s needs and usage patterns*, for example, clinicians who were less computer literate or trainees who may require layouts and clinician contexts that were more prescriptive (e.g., clinical scoring tools) compared to tech savvy and/or experienced clinicians. One physician stated, “the EHR probably should recognize who's using it… a lot of [clinical scoring tool] value is truly there for novice practitioners, and I probably have a bias 'cause I've been around for a long time and… I do think what a seasoned clinician needs from the EHR might be a little different than what a trainee needs from the EHR”. Lack of adaptation of EHRs to the user resulted in the inability to offload internal irrelevant processes onto the EHR^64^ (e.g., recalling where content is located within the EHR).

Secondly, the *absence of visual cues for navigating to relevant EHR data* for the specific patient encounter increased cognitive burden, “[C]hart review can be a pain sometimes. It's not the most pleasant thing to look at. It's a lot of just sort of linear stacked information and trying to decipher which […] clinic this note came from […]”. One nurse described generating cognitive aids (i.e., tools that reduced cognitive load by shrinking the signal-to-noise ratio) for colleagues in the chart so that critical elements could be prioritized^59^ during review:

“[E]verything is kind of written in the same font and stuff like that, so if there's something important… I'm going to put something there that they need to know. If they know nothing else about the patient, please just know this. I would just put that in bold, or things that they wouldn't readily see maybe […]”.

EHR design that is inconsistent with clinician mental models (i.e., broad conceptualization of an individual’s thought processes and how concepts interact with the world), which has shown to increase cognitive load,^60^ emerged as an additional perceived source of cognitive burden. *Inconsistency of the language used in the EHR with how clinicians discussed and thought about medical concepts and terminology* added to EHR cognitive burden and slowed clinicians down because they could not efficiently navigate to what they wanted to document in the chart: “[D]epending on who put [charting options] in there, it may not even be consistent with the terminology or definitions of how we were taught to do things… different from the textbook suggestion or the way I would describe it”, and “part of me wants to say, I feel like a non-medical person or non-medically trained person came up with [the labels]… this makes sense in my mind, but doesn't make sense in the person who created it”.

Research has revealed that excessive information and data—mediated by added cognitive load—are associated with hindered performance.^63^ *Overdocumentation (e.g., note bloat, redundant documentation) contributed to information overload and perpetuated information retrieval difficulties in the EHR.* Documentation burden, including lengthy notes and blowing extraneous data into the note led to cognitive burden because it rendered it impossible for clinicians to review and retrieve relevant clinical information from the patient chart:

“[T]he thing that makes it worse is the amount of stuff that gets automatically blown in from the previous records… that adds a lot of extraneous chatter into the electronic health record that you have to sift through to try to find the important things.”

Low perceived agency^61,62^ (i.e., reduction in “permissible actions”) has been linked to increased subjective mental effort. Clinicians perceived that *rule-based functionalities micromanaged clinician work.* In the EHR workflow, gatekeeping and constant reminders occurred at the least opportune moments and typically did not have sufficient context of what was critical at the point of care. For example, one physician stated, “it's true that sometimes I would write erroneous things on a paper chart, but the paper chart would never take my pen and say, ‘you can't write that’ or ‘you can't order that on this patient, they’re allergic to aspartame’”, while one nurse similarly stated, “Epic is very big on, ‘hey, you still have this running, are you sure you don't want to stop it?’ And it feels almost like [a] tattletale… when you click ‘stop infusion’, it says, ‘well, what time did you stop?’ ‘Well, what do you think? I stopped it 30 minutes from when I hung it up’, so that can be very frustrating”.
